# Temporal vision: measures, mechanisms and meaning

**DOI:** 10.1242/jeb.222679

**Published:** 2021-07-30

**Authors:** Kristian Donner

**Affiliations:** Molecular and Integrative Biosciences Research Programme, Faculty of Biological and Environmental Sciences, University of Helsinki, 00014 Helsinki, Finland

**Keywords:** Critical flicker frequency, Impulse response, Photoreceptor, Response latency, Retina, Motion vision

## Abstract

Time is largely a hidden variable in vision. It is the condition for seeing interesting things such as spatial forms and patterns, colours and movements in the external world, and yet is not meant to be noticed in itself. Temporal aspects of visual processing have received comparatively little attention in research. Temporal properties have been made explicit mainly in measurements of resolution and integration in simple tasks such as detection of spatially homogeneous flicker or light pulses of varying duration. Only through a mechanistic understanding of their basis in retinal photoreceptors and circuits can such measures guide modelling of natural vision in different species and illuminate functional and evolutionary trade-offs. Temporal vision research would benefit from bridging traditions that speak different languages. Towards that goal, I here review studies from the fields of human psychophysics, retinal physiology and neuroethology, with a focus on fundamental constraints set by early vision.

## Abstract (precis)

**Summary:** Simple measures of temporal vision such as the critical flicker frequency can be useful for modelling natural vision only if their relationship to photoreceptor responses and retinal processing is understood.

## Introduction

In animals with image-forming eyes, time and space are inseparably entangled in neural computations based on retinal light patterns that reflect both external and self-generated movement (Rucci et al., 2018). Temporal properties of vision are interesting mainly in connection with spatio-temporal analysis, and the visual system has evolved a rich set of tools for motion computations from the retina (Ölveczky et al., 2003; Chichilnisky and Kalmar, 2003; Mauss et al., 2017) to the brain (Wertheimer, 1912; Nijhawan, 2002; Schlag and Schlag-Rey, 2002; Levinthal and Franconeri, 2011; Wagemans et al., 2012). Yet, for reasons of experimental and analytical expedience, temporal performance has usually been studied in isolation. The temporal resolution of animals is commonly measured by the critical flicker (fusion) frequency (CFF) (see Glossary), but how can we relate this unnatural measure to the animal's performance in biologically relevant tasks? In dim light, where the sparse photon flux cannot carry high-frequency spatio-temporal information, long temporal integration may be a requirement for seeing anything at all. This is conventionally measured as the ‘critical duration’ (see Glossary) of stationary light pulses, defining a sharp integration or summation time. As known from photography, resolution and integration are opposite goals: sensitivity to stationary objects benefits from a long exposure, but the price is that moving objects become blurred or invisible (Lythgoe, 1979). Optimal trade-offs depend on the amount of light available, on the lifestyle of the animal and on the behavioural task. Investment in parallel neural pathways and physiological adaptation mechanisms represents evolutionary accommodation to this fundamental constraint.

In this Review, I consider how the traditional simple measures CFF and integration time are related to basic retinal mechanisms, the key to modelling performance in species-relevant tasks. This entails a tour from human psychophysics to photoreceptors and back through the retina to animal behaviour. The main focus is on vertebrates, with insects as an ‘outgroup’, acknowledging that arthropods have often led the way in vision research and continue to provide a rich treasure trove for the study of visual adaptations.

**Glossary**

**Centre-surround antagonism**

A ubiquitous principle of neural organization in peripheral sensory organs and the brain. In the retina, it concerns ganglion cells (GCs), bipolar cells and cones. It implies that an excitatory signal derived from the middle of the cell's receptive field (‘centre mechanism’) is opposed by a signal derived from a spatially wider but generally overlapping ‘surround’. Surround signals are mediated by horizontal and amacrine cells. At the GC, they may be excitatory but of opposite polarity (antagonistic), or purely suppressive (inhibitory). The centre and surround mechanisms of retinal GCs are generally roughly concentric.

**Critical duration**

A measure of the time over which the visual system or a neuron is able to sum responses to photons arriving at different times. Typically, the threshold light intensity (photons per area and time unit) for detecting a square-wave stimulus pulse is measured as a function of the duration of the pulse. Starting from a very brief pulse, the threshold intensity usually drops with increasing duration up to a limit, from which prolonging the pulse no longer lowers the threshold intensity. An equivalent measure can be obtained as the ratio of the threshold number of photons measured with a brief pulse to the threshold flux of photons (photons per second) measured with a long pulse.

**Critical flicker frequency (CFF)**

The high-frequency limit above which the response to a periodically modulated light cannot at any modulation amplitude be distinguished from the response to a steady field of the same mean luminance. The response may be the report of a human or animal observer, or an electrophysiological response of a neuron or eye. If the modulation is sinusoidal, the maximal modulation depth (see below) is 100% of the mean luminance, and the CFF is the frequency beyond which not even 100% modulation is ‘visible’.

**Eccentricity**

The retinas of vertebrates are often non-uniform, with different cell densities, sizes and proportions in different areas, so that different visual functions are optimized in different parts of the visual field. Humans and monkeys have a central fovea, specialized for high spatial acuity and lacking rods and blue cones but with a high density of slender red and green cones. The proportions and dimensions of cells change radially from the fovea. Eccentricity is the distance from the fovea expressed in degrees of visual angle.

**Filtering**

Filtering by a system means that some frequencies in a signal are reproduced less well than others (they are relatively attenuated). If high frequencies are attenuated, this constitutes low-pass filtering. If low frequencies are attenuated, this constitutes high-pass filtering. If both high and low frequencies are attenuated, this constitutes band-pass filtering. Filtering properties are directly related to the impulse response of the system (see Box 1).

**Flicker fusion**

The phenomenon that a temporally modulated light becomes indistinguishable from a steady light of the same mean luminance (Talbot's law) if the modulation amplitude is decreased or the frequency increased beyond some point.

**Fourier transform (FT)**

The FT translates a temporal (or spatial) light pattern into a function describing ‘how much’ the pattern contains of different (sinusoidal) frequency components. This function is a frequency spectrum. The FT of a photoreceptor's single-photon response describes how well it is able to transmit different temporal frequencies of light modulated in the linear response domain. Conversely, the single-photon or impulse response (see Box 1) can be recovered from the frequency response spectrum measured with periodic stimuli or white-noise stimulation (which contains an equal amount of all frequencies) by inverse FT (see Fig. 3F).

**Fractional sensitivity**

The fraction of the maximal light response (saturated response) of a photoreceptor elicited by one photoisomerization. For example, in photocurrent recordings from single photoreceptors, the maximal response amounts to turning off the entire light-sensitive current, and the fractional sensitivity is the fraction of the current turned off by one photoisomerization. The absolute sensitivity (response amplitude per photoisomerization) is the fractional sensitivity multiplied by the maximal response amplitude.

**Mean luminance**

Used in psychophysics to express the mean light level of a display, around which a periodic (flickering) stimulus is modulated. For a given pupil size and photoreceptor type, this allows calculation of the mean number of photoisomerizations [R*] per photoreceptor per second which is the measure of light intensity primarily used in this article. In experiments with non-periodic stimuli, the mean light intensity (to which stimulus pulses are added) is usually referred to as ‘background intensity’ (*I*_B_). For simplicity, the mean intensity of periodic stimuli [R* photoreceptor^−1^ s^−1^] is also denoted *I*_B_ in the present paper.

**Modulation contrast**

Contrast modulation implies that the stimulus light intensity is varied around a mean value (*I*_B_). Under sinusoidal modulation, the excursions up or down from the mean are equal, and the modulation contrast *c* is the modulation amplitude expressed as a fraction of *I*_B_ (sometimes called modulation depth). This is the Michelson contrast (*I*_max_−*I*_min_)/(*I*_max_+*I*_min_), as *I*_max_=(1+*c*)*I*_B_ and *I*_min_=(1−*c*)*I*_B_.

**Saccade**

A fast, large-amplitude shift of gaze to a new point in external space. In primates, saccades are produced by eye movements, whereas many animals perform saccadic movements of the head or body.

## Linear measures of temporal performance in human psychophysics and the primate retina

### The use of flicker in vision research

The CFF has great advantages and a deep history as a simple experimental measure of temporal resolution. It can be determined behaviourally by two-alternative choice experiments, where the animal has been trained to associate the percept of flicker with a reward, and electrophysiologically as the limit where the modulation response of a visual neuron, or of the entire retina or eye (the electroretinogram, ERG), can no longer be discriminated from noise (Piper, 1911; Dodt and Enroth, 1953). The ERG response of the intact eye *in situ* is an attractive proxy for behaviour, as it is fair to assume that visual signals not resolved by the eye cannot guide behaviour. The extensive early literature on flicker fusion (see Glossary) in humans and several invertebrates was reviewed by Selig Hecht and co-workers in the first of a series of five papers from 1932–1933 aiming to relate the CFF to Hecht's ‘photochemical theory of vision’ (Hecht and Wolf, 1932; Hecht and Verrijp, 1933a,b). The lasting popularity of the CFF is evident from two fairly recent compilations comprising, respectively, 34 (Healy et al., 2013) and 81 (Inger et al., 2014) species of vertebrates and arthropods. Hecht emphasized that the importance of the CFF as a temporal probe depends on its relationship to fundamental phototransduction mechanisms that constrain all vision. To elucidate this relationship, it is necessary to consider temporal modulation responses over the entire frequency range, where the CFF is just the high-frequency limit.

*Homo sapiens* is a nice model species for behavioural studies of vertebrate vision. Subjects are easy to train and respond patiently in experimental sessions lasting several hours. In the 1950s, Hendrik de Lange ushered in a new era in flicker studies by introducing linear-systems analysis of human vision (de Lange, 1952, 1954, 1957, 1958). He recorded flicker sensitivity as a function of frequency at different luminance levels and modelled the dominant low-pass filtering (see Glossary) properties by an electrical analogue, a chain of exponential delay stages (cf. Ives, 1922). When it became possible to record response waveforms of photoreceptors in arthropods and later in vertebrates, it was found that these could be described by similar linear models at low intensities or contrasts (DeVoe, 1962; Fuortes and Hodgkin, 1964; Baylor et al., 1974a,b; Baylor and Hodgkin, 1974; Daly and Normann, 1985).

**Fig. 1.**
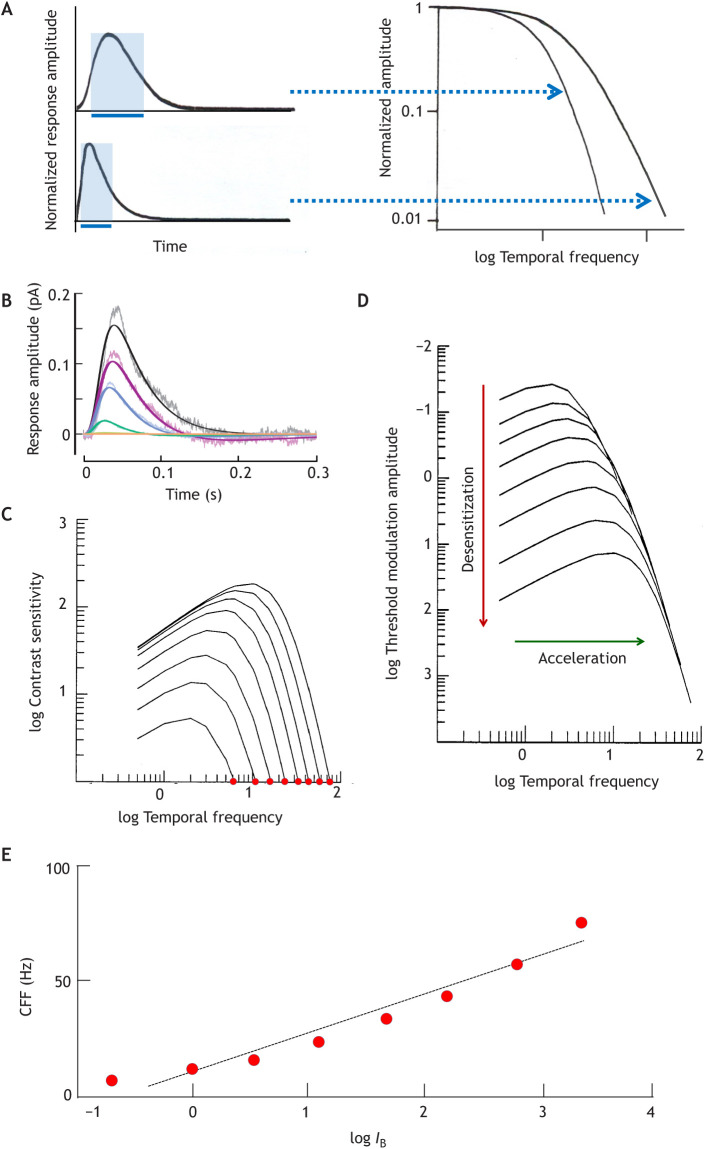
**Relationships between common temporal response measures in the linear response domain.** (A) Impulse responses (left) and corresponding temporal modulation transfer functions (TMTFs, right). The high-frequency limb of the TMTF extends to higher frequencies for the faster impulse response. The impulse response may be thought of as the ‘memory trace’ of a photon, to which the traces of subsequent photons can add. For some purposes, this can be expressed as a sharp interval, the summation or integration time *t*_i_, equal to the base (blue bars) of a rectangle with the same time integral (area) and the same height as the impulse response (shaded blue rectangles). For impulse responses of constant shape, *t*_i_ is proportional to the time to peak (*t*_p_). (B) Physiological impulse responses: single-photon responses of a macaque cone (linear-range flash responses divided by flash intensity [R*]) in darkness (largest response) and under four intensities of steady adapting background light (*I*_B_; from ∼10^3^ to ∼10^6^ R* s^−1^) (Angueyra and Rieke, 2013). The noisy traces are recordings, the smooth traces model fits. Temperature was 32°C. (C,D) Psychophysical TMTFs: foveal (cone-driven) flicker sensitivity functions at eight different mean luminances over a ∼3.5 log unit range. The families of curves were generated by the cone-inspired model of Rovamo et al. (1999) with the parameters that provided the best fit to their entire dataset (data points suppressed for clarity). Temporal frequency is in Hz. In C, sensitivity is the inverse of the threshold modulation contrast, whereas D plots threshold modulation amplitude (increasing downwards). The leftmost curve in C and the top curve in D correspond to the lowest *I*_B_. (E) Critical flicker (fusion) frequency (CFF) as a function of mean light intensity (log*I*_B_). Data points from C for log contrast sensitivity 0 (100% modulation, red dots) were fitted with a least-squares regression line (CFF=16.8log*I*_B_+10.5).

Fig. 1A illustrates the conceptual relationships between the basic temporal response measures according to linear-systems theory (Box 1). The other panels display physiological and psychophysical correlates recorded at several light levels (*I*_B_): single-photon responses from a primate cone and human foveal flicker sensitivity functions (temporal modulation transfer functions, TMTFs). In both, sensitivity and time scale change together as *I*_B_ rises over a certain range. The cone responses (Fig. 1B) peel off from a common rising phase at successively earlier times, coupling decreasing amplitude (desensitization) to shortening of the time to peak (*t*_p_; acceleration). With increasing mean luminance *I*_B_ (see Glossary), the human TMTFs extend to higher frequencies (further to the right, indicating acceleration), and although contrast sensitivity rises (higher peaks in Fig. 1C), larger modulation amplitudes (contrast × mean luminance) are needed for detection (desensitization: downward movement of curves in Fig. 1D). The relationship between desensitization and acceleration is such that the high-frequency limbs approximately converge on a common envelope (Kelly, 1961; for a critical look, see Rider et al., 2019).

**Box 1. Basic concepts of linear systems analysis**

A linear system can be characterized either by its impulse response (developing in time) or by its response to sinusoidal modulation (as a function of frequency). The two are mathematically interconvertible.

The impulse response is theoretically the response to an ‘infinitely brief’ stimulus pulse. The experimental counterpart in the vertebrate visual system is the dim-flash or, ideally, the single-photon response. If a linear system is completely noise-free (which the visual system is not), its temporal properties can be entirely derived from the waveform of the impulse response and, for example, signal gain plays no role. In reality, detection and resolution are limited by the signal-to-noise ratio (SNR) and depend on signal gain and sources of noise throughout the chain from photoreceptors via retinal circuits to the brain.

The response to sinusoidal contrast modulation as a function of frequency, the temporal modulation transfer function (TMTF), can be theoretically computed as the Fourier transform of the impulse response. Experimentally, it is the flicker sensitivity function. Monophasic impulse responses (such as the single-photon responses of photoreceptors) are associated with attenuation only of responses to high-frequency modulation (low-pass filtering, as shown in Fig. 1A). Band-pass filtering in psychophysical TMTFs, whereby responses to low-frequency modulation are also attenuated (Fig. 1C,D), arises from the interaction of antagonistic signals in the retina (Donner and Hemilä, 1996). The corresponding impulse response (not shown in Fig. 1) would be biphasic, with a late part undershooting the baseline.

The temporal persistence of the impulse response can for some purposes be expressed as a sharp time interval, the integration or summation time (*t*_i_, blue bars in Fig. 1A) (Baylor and Hodgkin, 1973). Such well-defined summation borders are intuitively helpful for thinking of time and space in terms of frame rates and pixels, and for transferring the discrete statistics of photon numbers to the world of neural signalling, but they have only limited validity and often become misleading (Field et al., 2019; Hemilä et al., 1998).

The position of each TMTF curve on the log frequency axis can be defined by a corner frequency *f*_c_, where sensitivity has decreased to a given fraction (here taken as 0.125) of the maximum. The increase in *f*_c_ with rising light level in Fig. 1C,D is well described by the equation log*f*_c_=0.17log*I*_B_+6.3 (*r*^2^=0.99) (Rovamo et al., 1999). A similar linear relationship (log*f*_c_∝*b*log*I*_B_) is seen in all published datasets on foveal flicker, from which a mean value of 0.14 is obtained for the proportionality constant *b* (variation range 0.10–0.17 in 7 subjects of de Lange, 1952; Kelly, 1961; and Roufs, 1972). In contrast, all datasets on foveal integration time *t*_i_ indicate a decrease with rising light level (log*t*_i_∝−*b*log*I*_B_) with mean *b*≈0.15 (0.10–0.18 in 9 subjects of Graham and Kemp 1938; Keller, 1941; Herrick, 1956; and Roufs, 1972) (see Donner et al., 1995). The reciprocity of *f*_c_ and *t*_i_ supports the linear assumption (Fig. 1A) that both reflect the kinetics (*t*_p_) of the impulse response: *t*_i_∝*t*_p_ and *f*_c_∝1/*t*_p_. Observing that a linear relationship of logarithms corresponds to a power-function relationship of the variables, we get:(1)
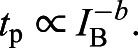
This type of relationship is universally observed in photoreceptors (see below and Table 1). For achromatic stimulation of the human fovea, *b*≈0.14–0.15.

**Table 1.**
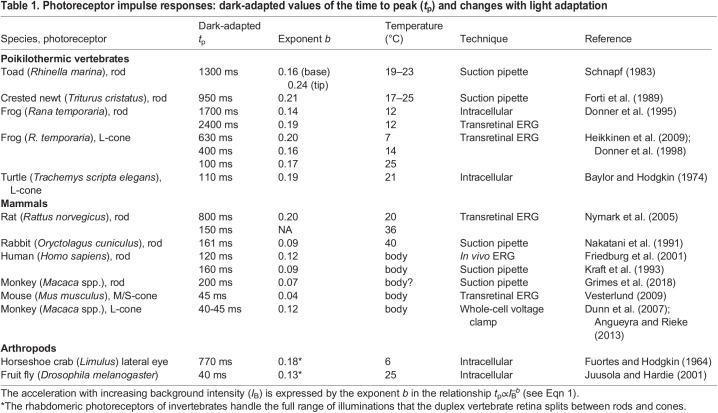
Photoreceptor impulse responses: dark-adapted values of the time to peak (*t*_p_) and changes with light adaptation

In a noise-free linear system, the high-frequency limb of TMTFs would have no limit: no ripples would be too small to be detectable, and there would be no CFF. In a real system, the response even to maximal (100%) modulation contrast (see Glossary) is submerged in noise above some frequency. In Fig. 1E, CFF values extracted from Fig. 1C are displayed as functions of log*I*_B_. Linear rather than logarithmic CFF values have been plotted, to honour the idea of an (approximately) linear CFF–log*I*_B_ relationship known as the Ferry–Porter law (Ferry, 1892; Porter, 1902; Ives, 1922; Hecht and Verrijp, 1933a,b). The small but systematic deviations from this ‘law’ in Fig. 1E may be due to several factors. Tyler and Hamer (1990, 1993) show that a rather strict linearity prevails in experimental conditions carefully controlled with respect to receptor input and eccentricity (see Glossary), but slope coefficients vary significantly (10–30 Hz/log*I*_B_) depending on eccentricity.

### Responses of cones and ganglion cells in the primate retina

Can cone responses account for the psychophysical flicker data? If so, they should accelerate with increasing illumination according to Eqn 1, with *b*≈0.14–0.15. We lack data on time scale adaptation in human cones, but monkey cone impulse responses (from Dunn et al., 2007) under a series of backgrounds *I*_B_≥1000 R* s^−1^ (photoisomerizations per second) are well fitted by Eqn 1 with *b*≈0.12. Moreover, in this *I*_B_ range, light adaptation in the primate retina is really due to the cone photoreceptors, whereas post-receptor mechanisms dominate at dimmer backgrounds (Dunn et al., 2007). Thus, in terms of acceleration with increasing *I*_B_, there is a satisfactory agreement.

Absolute (dark-adapted) time scales do not agree, though. The impulse response derived from TMTFs of the dark-adapted human fovea has *t*_p_≈125 ms (Rovamo et al., 1999). All recordings from monkey cones indicate kinetics that are more than twice as fast. Even foveal cones, where phototransduction and axonal properties are co-tuned for especially slow kinetics (supporting spatial acuity and high-fidelity signal propagation), have *t*_p_≈50–60 ms (Sinha et al., 2017; Bryman et al., 2020). Other monkey estimates fall in the range 35–55 ms (Schnapf et al., 1990; Schneeweis and Schnapf, 1995; Dunn et al., 2007; Angueyra and Rieke, 2013). In ERG recordings from the human eye under full-field stimulation, Friedburg et al. (2004) found cone *t*_p_≈15–20 ms. Instead, human foveal TMTFs agree well with older TMTF recordings from macaque lateral geniculate nucleus (LGN) cells (proxies for retinal ganglion cells, GCs) (Purpura et al., 1990; see also Lee et al., 1989). This indicates significant low-pass filtering downstream from cones, in line with results from the turtle retina (Baylor and Fettiplace, 1977). Surprisingly, Horwitz (2020) recently found little loss of high-frequency information between cones and the LGN or perception in the macaque. The difference might be at least partly explained by his use of stimuli better optimized for GCs and psychophysics (small drifting Gabor patterns).

A cautious conclusion is that human psychophysical sensitivities to high-frequency achromatic foveal flicker correlate closely with the responses of relevant retinal GCs/LGN cells, with a frequency dependence largely inherited from cones. This differs radically from the situation for chromatic flicker, where signals present in neurons at least up to the primary visual cortex are perceptually inaccessible (Lee et al., 1989; Gur and Snodderly, 1997).

### Flicker detection in the brain: no neural integration across cycles

The consensus that human TMTFs mainly reflect retinal filtering does not exclude modifications at the detection stage in the brain. Detector properties can be probed by adding dominant, purely temporal white noise to the flickering light stimulus, strong enough to swamp intrinsic early noise (Graham and Hood, 1992). An ideal signal/noise discriminator should then show no trace of the early filters, as it compares the signal at each frequency with noise at the same frequency, which has been passed through the same filters. By contrast, a detector looking at peak-to-trough amplitude will still reproduce the characteristics of the retinal filters, because it will compare the filtered signal with the total noise across all frequencies.

**Fig. 2.**
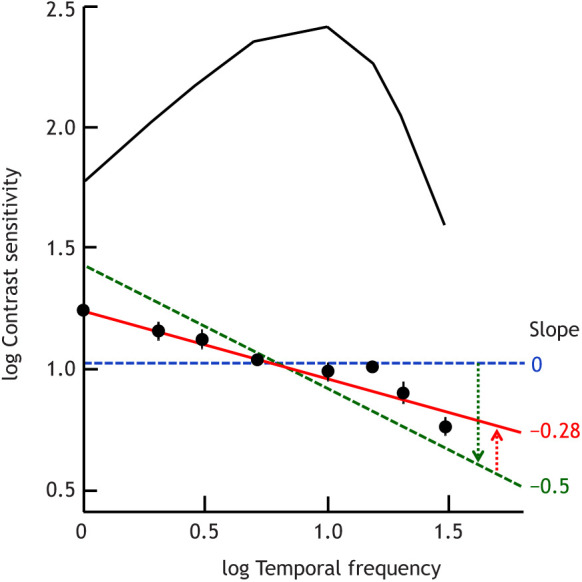
**Separating effects of retinal filtering and detection in the brain on psychophysical flicker sensitivity functions.** The symbols show means±s.d. of sensitivities measured with foveal flickering fields of five sizes (0.049–12.6 deg^2^) with added dominant purely temporal white noise (based on data from Rovamo et al., 2000). Temporal frequency is in Hz. The red line is a least-square fit. Shown for comparison is the familiar bandpass function recorded without added noise (black curve, cf. Fig. 1C), reflecting retinal filtering. The blue and green dashed lines are explained in the main text.

As shown in Fig. 2, adding dominant temporal white noise indeed wipes out the familiar bandpass shape of flicker sensitivity functions (black curve in the figure), leaving only a shallow decrease with increasing frequency (red line in the figure) (Rovamo et al., 1996, 2000). The features lost evidently represent retinal filtering. The deviation from the perfect frequency independence predicted for an ideal observer (blue dashed line with slope 0 in the figure) can be explained by the combined effects of two opposing factors: (i) matched-filter detection restricted to single cycles, favouring low frequencies, as the signal-to-noise ratio (SNR) is proportional to the square root of cycle duration; this produces a slope of −0.5 in a log–log plot (green dashed line in the figure) (Rovamo et al., 2003; cf. Barlow, 1958); (ii) probability summation across cycles, favouring high frequencies, which offer more single-cycle ‘trials’ in any given flicker epoch (Watson, 1979; Rovamo et al., 2003). The size of the latter effect depends on the steepness of the psychometric function relating the probability of detecting a single cycle to contrast (Watson, 1979). The effects of factors (i) and (ii) are graphically indicated in Fig. 2 (green and red arrow).

It should be noted that the data shown are means of sensitivities measured with stimulus fields covering a 256-fold area range, which all gave virtually identical results (Rovamo et al., 2000). Thus, the SNR was independent of spatial summation, i.e. the detection-limiting noise was indeed spatially homogeneous (100% correlated) up to 4 deg diameter. This means that there were no confounding effects of spatio-temporal ‘late’ noise (post-transduction retinal noise or brain noise) in these experiments.

The take-home message is that no true neural integration across cycles occurs even in the fairly resourceful human brain, indicating that conscious detection of periodic stimuli has not been evolutionarily important. Rather, it may be purposeful not to be distracted by predictably recurring signals, or even intrinsic retinal oscillations (Friedburg et al., 2004; Rangaswamy et al., 2003). In many species, responses to repeated presentations of the same stimulus are attenuated in the retina (Brown and Watanabe, 1965; Donner et al., 1991; Schwartz et al., 2007). Still, the monitoring of temporal regularity by some circuits is evident from the fact that small irregularities elicit strong ‘mismatch’ signals measurable by electroencephalography (EEG) or magnetoencephalography (MEG) in several sensory modalities (effectively used as research tools especially in the auditory system; e.g. Näätänen and Alho, 1997). An example from the visual system is the ‘omitted stimulus responses’ recorded in the brain of humans and lower vertebrates (Bullock et al., 1994), some of which have been shown to arise in the retina (Schwartz et al., 2007).

## Variation in the time scale of retinal responses between species

### Impulse responses of photoreceptors in different species: similar shape, different time scales

**Fig. 3.**
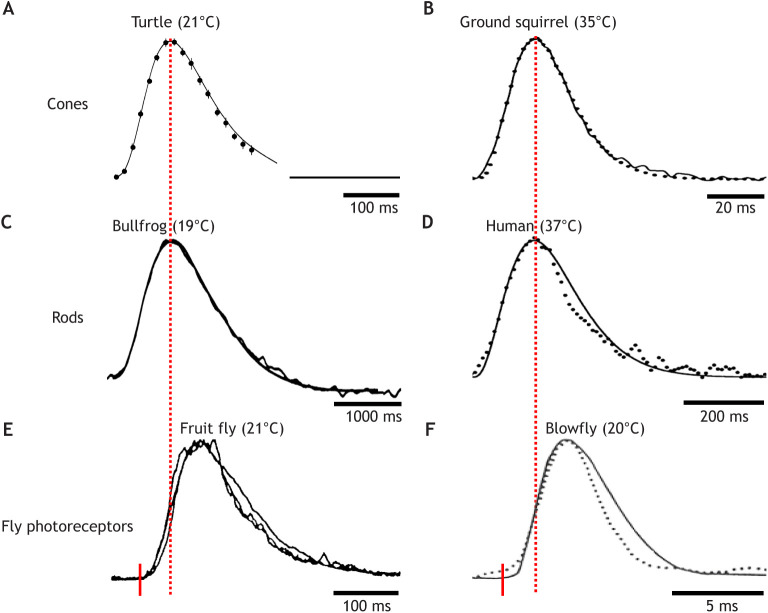
**Impulse responses of vertebrate cones and rods and fly photoreceptors have similar waveforms.** (A–E) Dark-adapted linear-range flash responses normalized to the same time to peak (*t*_p_), amplitude and polarity. Noisy traces are recordings, smooth traces model fits (see Box 2). Flash onset is at the start of each trace; the true time scales are indicated by the scale bars beneath the responses. Normalization of the fly responses (E and F) to the vertebrate responses was done as if the flash had occurred at the red tick, i.e. disregarding the ‘dead-time’. (A) Reptile L-cone voltage (*Trachemys scripta elegans*) (Baylor et al., 1974a,b). (B) Mammalian L-cone current (*Citellus lateralis*) (Kraft, 1988). (C) Amphibian rod current (*Lithobates catesbeianus*) (Donner et al., 1990a). (D) Human rod current (Kraft et al., 1993). (E) Fruitfly photoreceptor current (*Drosophila melanogaster*, late-stage pupa of *Shaker* mutant) (Hardie, 1991). (F) Blowfly photoreceptor voltage (*Calliphora erythrocephala*), moderately light adapted (Weckström et al., 1988). Continuous line, dim-flash response; dotted trace, impulse response obtained by inverse fast Fourier transform (see Glossary) from the recorded TMTF.

The properties of photoreceptor responses ultimately constrain the visual temporal information available for any task. The monophyletic origin of vertebrate phototransduction is reflected in a remarkably constant waveform of the impulse (dim-flash) response of cones and rods (Fig. 3A–D), in spite of 50-fold or, depending on the temperature, >100-fold differences in absolute time scale (scale bars beneath each response in Fig. 3). Even more remarkably, impulse responses of rhabdomeric receptors have converged on a very similar waveform, although based on entirely different mechanisms (Howard et al., 1984; Hardie, 1991; Fain et al., 2010). Apart from being depolarizing instead of hyperpolarizing, the fly impulse response (Fig. 3E,F) is not even a single-photon response, but shaped by the latency and amplitude distribution of many quantal ‘bumps’. Otherwise, vertebrate cones and fly photoreceptors respond very similarly, not only to flashes or sinusoidal contrast modulation but also to time series of intensity variation scanned from natural environments. Both use non-linearities and fast gain controls to compress and normalize the skewed natural intensity distributions and make information-efficient use of their limited dynamic signalling ranges (van Hateren and Snippe, 2006) – based on different mechanisms but with similar results.

**Box 2. The shape of vertebrate photoreceptor responses**

**Fig. 4.**
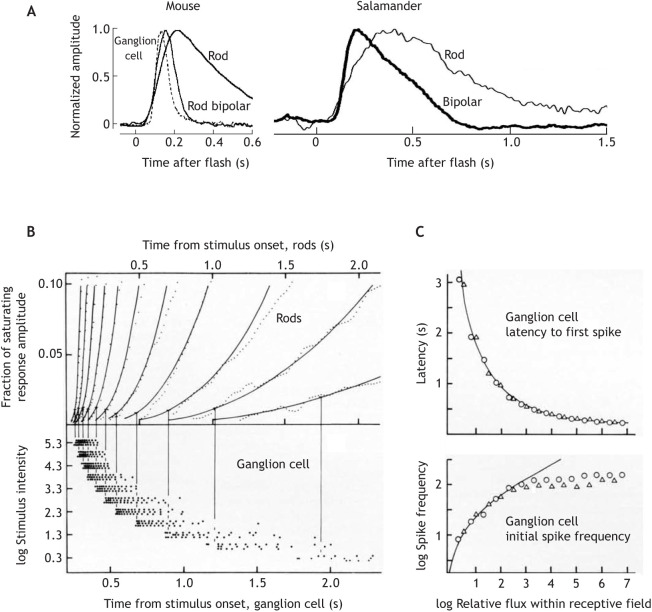
**The importance of the leading edge of photoreceptor responses: retinal transmission of rod signals and readout by ganglion cells.** (A) Responses to dim light flashes recorded in the dark-adapted mouse and salamander retina, showing high-pass (or band-pass) filtering of the rod signal during retinal transmission to bipolar and ganglion cells. Left: current responses of a mouse rod, rod bipolar and ON-ganglion cell (GC) (body temperature). Right: current responses recorded simultaneously in a salamander rod and ON-bipolar cell (*Ambystoma tigrinum*, room temperature). The rod responses have been flipped vertically to agree in polarity with those of the higher-order cells. After Field et al. (2005). (B) Relationship between rod and GC responses in the dark-adapted frog retina (*Rana temporaria*, 11°C) to steps of light of the same 11 intensities (from 2 to 2×10^5^R* rod^−1^ s^−1^ as indicated on the lower ordinate). Top: the leading edge of rod responses recorded by transretinal ERG (noisy dotted traces), fitted by a family of model responses (smooth curves, see Box 2). Bottom: spike responses of a class 3 (ON–OFF; Lettvin et al., 1959) GC to a small stimulus spot (much smaller than the retinal field, RF), extracellularly recorded in the eyecup. Each dot marks one spike; each line of dots is the response to one stimulus presentation. Vertical lines mark the mean latency of three responses to the same intensity. The family of GC responses has been shifted leftwards by 200 ms relative to the rod responses to account for a constant rod-to-GC transmission delay. (C) Top: latency from the onset of a step stimulus to the first spike. Bottom: initial spike frequency (s^−1^) over the first four spikes, recorded in another dark-adapted class 3 GC. Both are plotted as functions of log photon flux [R* s^−1^] summed over the RF, relative to threshold flux. Triangles, stimulus spot much smaller than the RF; circles, spot larger than the RF. The smooth curves are model functions calculated on the assumptions that (1) the first spike occurs when the rod response linearly summed over the RF has reached a criterion amplitude, plus a constant ‘transmission delay’; (2) the initial spike frequency is determined by the steepness of the leading edge of the summed rod response over a short interval after that. After Donner (1989).

Phototransduction in cones and rods defines the dynamics of their single-photon responses, which set ultimate constraints on temporal vision. All visual information available for image processing has been filtered through these (disregarding intrinsically light-sensitive ganglion cells, GCs; e.g. Fernandez et al., 2018). The underlying mechanisms are understood on the molecular and cellular level in exceptional quantitative detail (Lamb and Pugh, 1992; Hamer et al., 2005; Krispel et al., 2006; Lamb, 2013; Arshavsky and Burns, 2014; Lamb and Kraft, 2016), but in the present context, we are concerned only with a description of the output, the electrical response. In the early 1970s, Alan Hodgkin together with Denis Baylor and Trevor Lamb in Cambridge developed a class of linear, low-pass filter-chain models, loosely identified with hypothetical chemical reaction cascades, to describe their pioneering single-cell recordings from turtle cones. These models were not really helpful for unravelling the molecular processes of phototransduction, but the equations provide simple yet accurate phenomenological descriptions of response waveforms *r*(*t*). Fig. 3A (from the original work of Baylor et al., 1974a,b) shows a fit of the ‘independent activation’ version of the model to an averaged ‘flash’ response:
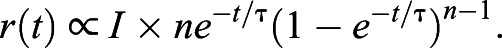
This equation integrated over time (*t*) gives the ‘step’ responses used for fitting the rising phase of rod responses in Fig. 4B and for the latency and frequency functions in Fig. 4C. The parameters are (1) the number of stages in the filter chain (*n*) (3–7), defining how sharply the response rises and falls, and (2) the time constant τ, defining the absolute time scale of the response. Both the time to peak (*t*_p_) and the integration time (*t*_i_) are directly proportional to τ. The response scales linearly with light intensity (*I*) at small amplitudes (small ‘linear-range’ responses as well as early phases of larger responses), before effects of saturation or adaptation set in. Other phenomenological models differ mainly in the decay phase of the responses, some describing biphasic responses (Baylor et al., 1974a,b, 1983; Schnapf et al., 1990; Angueyra and Rieke, 2013).

### Effect of light level

Species differences of vertebrate photoreceptors arise from different isoforms of transduction molecules and differences in cell morphology (Lamb, 2013). When comparing time scale measurements, however, two external variables that modify response kinetics physiologically must be taken into account: light level and temperature. The former affects all animals, the latter mainly poikilotherms. With rising light levels, photoreceptors encounter both the necessity to desensitize in order to escape saturation and the opportunity to improve temporal resolution, making use of the increased information carried by the photon flux. Decreasing *t*_p_ over moderate (∼2–3 log unit) ranges of increasing illumination is a universal phenomenon that has evolved independently in both ciliary and rhabdomeric photoreceptors (Howard et al., 1984; Fain et al., 2010). Eqn 1 provides a useful description in a wide spectrum of photoreceptors, although the exponent *b* varies (Table 1), as does the relevant *I*_B_ range. In vertebrate (but not insect) photoreceptors, acceleration and desensitization are typically mechanistically coupled, as the response shut-off gets faster (Fig. 1B) but the rising (activation) phase remains constant. The lowest and highest illumination ranges are exceptions. At low *I*_B_ levels, cone response amplitude may stay constant or even increase, although *t*_p_ decreases (frog: Donner et al., 1998; Heikkinen et al., 2009; monkey: Dunn et al., 2007). At very high illumination levels, in contrast, the decrease in *t*_p_ levels off and is even reversed while sensitivity continues to drop (turtle: Baylor and Hodgkin, 1974; frog: Donner et al., 1998). A secondary slowing down of cone-driven responses at high illumination levels is also evident in the behavioural CFF of birds (Lisney et al., 2011; Boström et al., 2016).

Rods have a much greater capacity for both sensitivity and time scale adaptation than often thought. Frog rods show acceleration-coupled desensitization over ∼5 log units of *I*_B_ (Donner et al., 1995). Possibly, rods may escape complete saturation even under very bright continuous illumination (mouse: Tikidji-Hamburyan et al., 2017). There is a substantial mesopic range where both rods and cones are active (human psychophysics: Conner and MacLeod, 1977; monkey rods: Grimes et al., 2018).

The convergence of signals from large numbers of photoreceptors on GCs, especially in the mammalian retina (Sterling et al., 1988), necessitates the activation of post-receptoral gain control mechanisms at light levels where single photoreceptors do not yet ‘see’ a need to light adapt (e.g. Shapley and Enroth-Cugell, 1984; Donner et al., 1990b; Brown and Rudd, 1998). In the primate retina, the post-receptoral gain decreases in the low photopic range are also associated with response acceleration (Dunn et al., 2007). With respect to post-receptoral time scale adaptation in primate rod vision, there is at present no consensus. Grimes et al. (2018) concluded that GC responses in the macaque retina accelerate in tandem with rod voltage responses, and that the rod signals are mediated almost exclusively by a single pathway (the rod bipolar pathway). In contrast, Stockman et al. (2010) concluded that there is significant post-receptoral acceleration in the lowest scotopic range in humans. Moreover, with rising light levels, the human rod-mediated CFF shows a complex dependence on *I*_B_, which has been attributed to the interference of signals mediated by two kinetically differing rod pathways (Conner and MacLeod, 1977; Conner, 1982; Sharpe et al., 1989).

### Effect of temperature

Temperature is the other physiological variable that strongly affects the time scale of photoresponses. Warming acts differently from increasing illumination. It accelerates the entire response, including the rising (activation) phase, and while it reduces fractional sensitivity (see Glossary), it increases the saturating response amplitude in a certain temperature range (toad rods: Baylor et al., 1983; Lamb, 1984; frog rods: Donner et al., 1988; frog cones: Heikkinen et al., 2009; rat rods: Nymark et al., 2005). This is significant, because the timing of visual events depends on the dynamics of the rising phase (see below). Mechanistically, activation speed has been linked directly to the diffusional (thus temperature-dependent) rate of protein–protein encounters in the photoreceptor membranes (mouse rods: Calvert et al., 2001).

Dark-adapted responses of amphibian photoreceptors typically accelerate by 2- to 3-fold per 10°C temperature rise (*Q*_10_=2–3). When mammalian photoreceptors are cooled from body temperature, they decelerate even more steeply (*Q*_10_≈4) (Nymark et al., 2005). In natural conditions, photoreceptor temperature in mammals and birds is largely stabilized by massive choroidal blood flow (Bill et al., 1983; Parver, 1991). Cooling would make vision slower and warming would increase thermal noise (cf. Aho et al., 1993a). Several big oceanic fish predators actively heat their retinas to increase the speed of vision. In the swordfish (*Xiphias gladius*), keeping the retina at >20°C has been estimated to improve temporal resolution by more than 10-fold compared with what it would be at the temperature of its hunting grounds in the cold deep sea (Fritsches et al., 2005).

TMTFs intracellularly recorded in photoreceptors of the blowfly *Calliphora vicina* indicate *Q*_10_≈3.0 in the dark-adapted state and *Q*_10_≈1.9 in the light-adapted state. At 34°C, its light-adapted photoreceptors are the fastest recorded in any species (*t*_p_≈5 ms: Tatler et al., 2000). The lower *Q*_10_ in the light-adapted state is in line with findings from vertebrate rods and cones, which show that effects of increased illumination and warming are only partly additive (Nymark et al., 2005; Heikkinen et al., 2009).

### The CFF as a measure of the speed of vision in different species

The CFFs of eyes can be relatively easily determined by ERG, but even for known response waveforms they cannot be automatically converted into photoreceptor kinetics. Nonetheless, the CFF remains a useful index of inter-species differences in the speed of vision.

A general problem with much CFF data, though, is the inadequate documentation of light level and temperature. Therefore, the conclusions that can be drawn from the 81-species compilation of Inger et al. (2014) (their table 3) remain on a rather general level. Insects have faster vision than vertebrates, and within these groups, diurnal, fast-moving and predominantly flying species have the highest CFFs. Healy et al. (2013) analysed data from 34 vertebrate species and found some support for the hypothesis that small size and high metabolic rate correlate with high temporal resolution. It might be worthwhile to do similar studies with sharper focus, e.g. in relation to action radius and body inertia.

Studies of judiciously delimited clades and ecological gradients may better elucidate relationships between adaptations and constraints (cf. Jourjine and Hoekstra, 2021). To take a few examples, Jenssen and Swenson (1974) measured the CFF of seven species of *Anolis* lizards in an optomotor rotating-drum paradigm, finding a clear correlation between preference for brighter habitats and higher CFF. Yet, Steinberg and Leal (2016) found no significant difference in motion detection between six *Anolis* species with different habitat preferences (two of which were the same as in Jenssen and Swenson, 1974), underscoring the task dependence of temporal performance. Frank (1999) measured the CFF of 8 species of mesopelagic crustaceans by ERG. The expected general trend of decreasing CFF with increasing depth of habitat was broken by two deep-sea outliers with high CFF, putatively explained by the availability of light from bioluminescence in the deep-sea habitat. Eight deep-sea benthic crustaceans studied by Frank et al. (2012) at 7°C had rather varying CFFs, with the 4 Hz of the isopod *Booralana tricarinata* claimed by the authors as the lowest measured in any species. This may be literally true, but in fact the CFF of toad rods (Nowak and Green, 1983) corrected to the same temperature would be about 2 Hz. Ryan et al. (2017) determined the CFF of the (rod-dominated) ERG of 5 shark species, finding a clear difference between, on the one hand, two tropical species not known to go deeper than 85 m (CFF≈40 Hz) and, on the other hand, three species foraging at much greater depths (CFF≈30 Hz) (all measured by ERG at room temperature). Cephalopod CFFs seem to fall in the same range, around 30 Hz (Bullock and Budelmann, 1991).

**Fig. 5.**
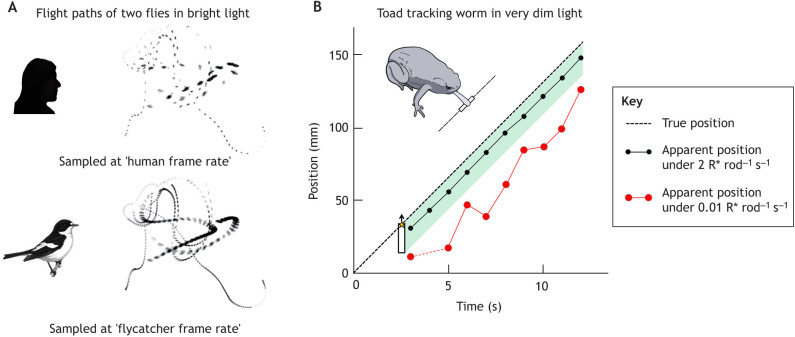
**Prey-tracking and motion extrapolation.** (A) The flight paths of two blowflies sampled at frame rates of 40 and 120 Hz, corresponding to human and flycatcher CFF at a fairly high luminance level (after Boström et al., 2016). (B) Prey capture in very dim light by the toad *Bufo bufo* (temperature 15°C). White worm dummies illuminated from above moved at constant speed against a black background. The dashed straight line shows the actual position of the worm's head as a function of time. The dots connected by lines indicate the apparent position of the worm's head based on the information that the retina sends to the brain at each moment. These have been calculated from the (variable) latencies of single responses of a GC stimulated repeatedly with the retinal image of the worm at two illumination levels: ∼1% of full moonlight (black) and 200-fold lower illumination (red). The green-shaded area is a zone of ‘acceptable’ mislocalizations, where snaps would still hit the worm.

Some diurnal birds have the highest CFF among vertebrates. In the 1950s, Dodt and Wirth (1953) showed by ERG that pigeons may resolve up to 140 Hz. For comparison, the highest value measured in any mammal is 108 Hz in light-adapted ground squirrels (Tansley et al., 1961) (see the fast dark-adapted cone response in Fig. 3B). Over the last decade, Ödeen, Kelber and co-workers have published a series of elegant behavioural CFF studies on birds with attention to ecology and taxonomy (Rubene et al., 2010; Lisney et al., 2011; Boström et al., 2016, 2017; Potier et al., 2020). Chicken do not have a very high CFF, but interestingly, an old non-selected breed has higher resolution than modern commercial laying hens (Lisney et al., 2011). Insectivorous passerines have high CFFs, with one individual pied flycatcher (*Ficedula hypoleuca*) reaching 146 Hz (Boström et al., 2016; see Fig. 5A), whereas the budgerigar (*Melopsittacus undulatus*; also a small bird, but feeding on seeds and slow-moving insects) does not reach more than ∼90 Hz (Boström et al., 2017). Among diurnal raptors, the peregrine falcon (*Falco peregrinus*), which catches fast-moving, manoeuvrable prey in flight, has higher CFF (129 Hz) than two species catching slower prey (Potier et al., 2020).

Day-active flying insects such as flies, bees and dragonflies generically have the highest ocular CFFs of all animals (up to 240 Hz) (Inger et al., 2014). Although a value of 400 Hz has been recorded by ERG in the infra-red-sensitive beetle *Melanophila acuminata* (Hammer et al., 2001), this is not a true visual response but a heat response probably mediated by TRP channels. The record for fast vision is set by light-adapted blowflies (Autrum, 1950; Tatler et al., 2000), which obviously serves them well when performing sharp manoeuvres in confined spaces and neck-breaking chasing feats, as recorded by Land and Collett (1974) (see also Fig. 5). The price is low sensitivity in dim light, when extensive temporal summation would be needed (fortunately silencing them at night in our bedrooms). The matching of the speed of vision to different average light levels in diurnal versus nocturnal dipteran species also involves tuning the time constant of the photoreceptor membranes by appropriate mixes of potassium conductances (Laughlin and Weckström, 1993). Adaptations for ‘fast’ bright-light vision entail adaptations for high information rates and are energetically costly in the depolarizing rhabdomeric receptors, where they are associated with large ion fluxes (Laughlin et al., 1998; Niven et al., 2007; Niven and Laughlin, 2008; Fain et al., 2010). Moreover, information is energetically more expensive in higher- compared with lower-performance cells, establishing a ‘law of diminishing returns’ on evolutionary investments in increased information capacity (Niven et al., 2007) [the energy budget is very different in light-hyperpolarizing (vertebrate) receptors; Okawa et al., 2008]. Insect eyes offer amazing examples of evolution tinkering with established solutions, exploring routes to new optima that may be diametrically opposite to the original ones. The transformation of the diurnal eye design of bees and wasps for a nocturnal lifestyle in *Megalopta* and *Xylocopa* (Central American and Indian bees) has involved the slowing-down of photoreceptor responses together with changes in optics and neural summation in downstream circuits, all serving to enhance sensitivity at the expense of resolution (Warrant, 2008; Frederiksen et al., 2008; see also Stöckl et al., 2016, for the nocturnal lepidopteran *Deilephila elpenor*).

It may finally be noted that flicker sensitivity has become a research subject in its own right motivated by the enormous spread of flickering fluorescent and LED light sources. In fact, the express purpose of Inger et al. (2014) in their CFF data compilation was to provide a basis for assessing to what extent the actual flicker of artificial lighting may affect animals, besides more obvious parameters such as occurrence, timing and spectral composition. High-frequency flicker above the conventionally accepted human CFF (∼60 Hz) may be stressful for production and laboratory animals, especially birds, and even for humans (Kuller and Lalke, 1998; Inger et al., 2014; Gladin and Kavtarashvili, 2021). Another line of applied flicker research has the goal to find parameters that may specifically attract (for traps) or repel (for home lighting) obnoxious insect species (Chu et al., 2006; Barroso et al., 2015; Wilson et al., 2021).

## Timing of visual events

### Reading the rising phase of photoreceptor responses

It has often been pointed out how smart the vertebrate eye is (Lettvin et al., 1959; Gollisch and Meister, 2010). This is true even in the ‘linear’ response domain. Although for some purposes it is practical to model temporal integration as shown in Fig. 1A by a sharp integration time *t*_i_ within which photons arriving at different times are pooled indiscriminately (Bloch, 1885), this works only for the task by which it is measured (e.g. the critical duration). Dim light pulses of different durations ≤*t*_i_ that are equally (barely) detectable can still be discriminated with high reliability (Zacks, 1970). Even the single-photon response of a dark-adapted primate rod enables an ideal detector to reach several times higher resolution of photon arrival time than suggested by a digital ‘frame rate’ based on the integration time of human rod vision, or of the rod itself (Field et al., 2019). Such high temporal precision can be achieved by focusing on the early rising phase of the rod response, which is much less variable than the later parts (Field and Rieke, 2002; Doan et al., 2006), and this is exactly what the retina does. Fig. 4A shows how the rod response in mammals and amphibians is high-pass filtered in the first synapse, so that bipolar cells respond mainly to the leading edge of the response (Armstrong-Gold and Rieke, 2003; Field et al., 2005; cf. dogfish: Ashmore and Falk, 1980; turtle: Schnapf and Copenhagen, 1982). Murphy and Rieke (2006) studied mouse GC spike responses to repeated presentations of the same epoch of randomly modulated light at low scotopic intensity, finding crisp spike bursts of remarkable reproducibility. The standard deviation across trials of the timing of the first spike in the bursts amounted to just a few per cent of the duration of the dim-flash rod response.

### Response latencies to supra-threshold steps

The easiest timing data to interpret are response latencies to square-wave light pulses. Such stimuli are not at all unnatural, as the retinal images of contrast borders easily traverse GC receptive fields (RFs) in milliseconds, producing sharp incremental or decremental contrast steps. This is also true under self-generated movements, most obviously in connection with saccades (see Glossary). Gollisch and Meister (2008) showed that the high spatio-temporal precision of GC spiking patterns recorded by multielectrode arrays in the salamander retina after saccadic movements would in principle allow fast recovery of the spatial structure of the scene based on the relative latencies of the first spikes. In the human eye, even microsaccades (small involuntary eye movements during fixation) may sweep a border across a 1 deg diameter RF in 10 ms (Martinez-Conde et al., 2004).

Fig. 4B shows the relationship between rod and GC responses in the dark-adapted frog retina to steps of light over a wide intensity range. The GC spiking discharge always begins at an approximately constant rod amplitude, corresponding to ∼1% of the saturated response amplitude. The criterion is reached at successively earlier times with increasing light intensity. Latency shortens steeply over 2–3 log units from threshold, and then asymptotically approaches an irreducible rod–GC transmission delay. Even this very slow photoreceptor (*t*_p_=3.6 s at 11°C) can support reaction times of a few hundred milliseconds at higher intensity or contrast. Latency variation between trials is <1% of *t*_p_ over most of the range. Further, stimulus intensity and area are interchangeable in their effect on latency: latencies to different-sized stimuli coincide when plotted as functions of photon flux within the GC RF (Fig. 4C), as expected for a signal that depends linearly on both area (Barlow, 1953) and intensity (see Box 2).

Latency functions of the form shown in Fig. 4C also give good descriptions of human reaction times in both scotopic and photopic states, with time parameters appropriate for human vision in the respective state of adaptation. The precise signal transformations en route from photoreceptors to the human subject's pushing of a button remain unresolved, but the correlations support the idea that the intensity dependence is inherited from the leading edge of photoreceptor responses. Specifically, the linear summation over contiguous areas (Vassilev et al., 2002; Donner and Fagerholm, 2003) implicates a signal determined by the early linear part of receptor responses.

Timing also shapes signal integration in the retina. A striking example is the induction of flicker colours by achromatic patterns on discs rotating at certain velocities, ascribed to phase shifts between antagonistic signals from blue-sensitive cones (phase-lagging) versus green- and red-sensitive cones (e.g. Schramme, 1992). Classical models of movement selectivity explicitly involve relationships between motion velocity and response timing (Reichardt, 1961; Borst and Egelhaaf, 1989; Barlow and Levick, 1965; Sivyer et al., 2010; Mauss et al., 2017; Hanson et al., 2019). The effects of centre-surround antagonism (see Glossary) in GC RFs (both linear and non-linear) critically depend on temporal relationships of the interacting signals, determined by stimulus-dependent components and relative delays (Nye and Naka, 1971; Donner, 1981a,b; Donner and Hemilä, 1996; Borghuis et al., 2018). The integration of centre-surround inputs at the level of GC RF subunits (Turner et al., 2018) underscores the multi-layered complexity of temporal processing of spatial contrast at different scales. Retinal latency relationships also affect the centre-surround interaction of contrast patterns in the brain (Kilpeläinen et al., 2007).

### Response speed correlates with the scaling of stimulus magnitude

Initial spike frequency, a putative neural magnitude code, is closely coupled to response latency in certain classes of GCs (Fig. 4B,C). With increasing stimulus intensity, latency shortens, and successively earlier and steeper segments of the photoreceptor response are read for this code. Although the early rod signal scales linearly with light intensity, this readout produces a non-linear, compressive and in principle non-saturating intensity code (e.g. over a population of GCs, although single GCs saturate). Such functions fit data on human brightness scaling (Stevens and Stevens, 1963; Marks and Stevens, 1966; Mansfield, 1973) at different adaptation levels, when time scale changes following Eqn 1 are observed. Especially persuasive is the good fit to data describing flash intensities that produce equal supra-threshold brightness percepts under different backgrounds (Djupsund et al., 1996; see data of Whittle and Challands, 1969). The psychophysical magnitude scaling of positive and negative contrast is largely symmetrical (Burkhardt et al., 1984, 1987; Burkhardt and Gottesman, 1987), suggesting that the OFF-pathway may read the early falling phase of photoreceptor responses to light decrements in a similar manner to that by which the ON-pathway reads the early rising phase of increment responses.

### Light decrements and negative contrast

The split of the visual information into ON and OFF excitation in the first synapse is ubiquitous in vertebrate retinas, even being present in cyclostomes (Ellis et al., 2020). It is reflected in the retinal output as classes of GCs that increase their firing in response to either brightening or darkening, or both. Sudden dimming of parts of the visual field, or increases in the visual angle subtended by a dark looming silhouette, signals imminent threat of predation or collision. Such stimuli elicit fast escape or freezing reactions in both vertebrates and insects, driven by partly resolved sensory-motor circuits (frog: Grüsser and Grüsser-Cornehls, 1968; mouse: Münch et al., 2009; Yilmaz and Meister, 2013; Kim et al., 2020; pigeon: Wu et al., 2005; fruit fly: Gibson et al., 2015; Zacarias et al., 2018). To light-hyperpolarizing photoreceptors, darkening appears as a classical excitatory stimulus that depolarizes cells. Interestingly, several invertebrates use ‘unorthodox’ light-hyperpolarizing receptors for alert responses to shadows (Hartline, 1938; Leutscher-Hazelhoff, 1984; Wilkens, 2008). In vertebrate rods and cones, darkening leads to increased glutamate release, which excites second-order cells (OFF-bipolars) directly via ionotropic glutamate receptors, whereas excitation of ON-bipolars by light (glutamate decrease) requires a postsynaptic transduction cascade controlled by a metabotropic glutamate receptor. Although remarkably fast among G-protein cascades, it still causes a delay (e.g. Martemyanov and Sampath, 2017). Indeed, GC OFF-responses have generically shorter latencies than ON-responses (turtle: Baylor and Fettiplace, 1977; frog: Donner and Grönholm, 1984). In mammals, this primary OFF-advantage may be relatively less important compared with other differences between the pathways. Ala-Laurila et al. (2011) recorded input currents of macaque GCs while stimulating single cones with randomly modulated voltage or light. They found that the transfer of signals driven by injected voltage was indeed significantly faster to OFF- than to ON-GCs, but that the difference was slight for light-driven signals.

Although the general usefulness of splitting pathways is beyond doubt (e.g. Gjorgjieva et al., 2014), unravelling how information from ON and OFF channels is integrated for building representations of the environment remains a major challenge in mammalian vision research. It is clear that the channels are not always used optimally from an information-theoretical viewpoint. For example, photon detection by mice in darkness would be most sensitive if based on gaps in the firing of OFF-GCs, but this information is not used. Instead, behaviour follows less sensitive ON-GCs that signal photons by increases in spiking (Smeds et al., 2019).

## Motion detection and extrapolation

### Resolving temporal order in space

As seen above, the leading edge of photoreceptor responses can support timing precision more than an order of magnitude higher than suggested by measures such as *t*_p_ or CFF. Humans can discriminate between flickering and steady artificial bright lights up to 1–2 kHz (Roberts and Wilkins, 2013), if fast eye movements convert the flicker into a discrete spatial sequence of flashes on the retina. Such ‘phantom arrays’ (Hershberger and Jordan, 1998) may be elicited, for example, by LED car tail lights around saccades.

In natural vision, high temporal resolution in space supports motion detection. Westheimer and McKee (1977) found that when stationary line stimuli with sharp onset were asynchronously presented at two adjacent points on the human fovea, their temporal order could be correctly identified down to an asynchrony of ∼3 ms. This asynchrony evoked a motion percept when the lines were parallel, but the high temporal resolution was not contingent on motion perception, as the onset order of two orthogonal lines forming a cross was equally well resolved. Motion decoding in the brain is thought to be based not just on the timing of the first spikes elicited by an object moving over an ensemble of GCs but also on the temporal structure of longer spike responses, whose reproducibility and similarity in adjacent GCs of the same type enable remarkably precise correlations of relative spike timings between cells (Chichilnisky and Kalmar, 2003; Borghuis et al., 2019). Correlation of spikes with high temporal (∼10 ms) precision is predicted to also improve the fidelity of motion perception on much slower time scales (Butts et al., 2007). Performance may be further improved by population coding (Frechette et al., 2005).

### Prey capture and motion extrapolation

Capturing moving prey requires accurate spatio-temporal localization based on visual information that reaches the brain after significant neural delays. It is obvious that some predictive computation (extrapolation of motion) is needed to correct for the delays (Nijhawan, 1994). Nonetheless, the extrapolation accuracy will depend on how well the preceding trajectory is resolved in space and time. Fig. 5A is an intuitive visualization (from Boström et al., 2016) of the relative advantage afforded by the fast vision of a pied flycatcher compared with a human in resolving the flight paths of flies. Note, however, that the ‘video frame rate’ analogy is misleading with regards to the actual mechanisms of motion processing (see above).

Fig. 5B illustrates an experiment on a toad, where the 100-fold slower time scale facilitates quantitative analysis of the relationship between behaviour and retinal signals (after Aho et al., 1993b). At the lowest light level near the sensitivity limit of vision, snaps based on the retinal information at each moment (red dots) would always miss the moving ‘worm’. In fact, the toad often hit the worm, which suggests a remarkable capacity for motion extrapolation. Similar results have been obtained in salamander by Borghuis and Leonardo (2015). The toad is poised between the need to increase temporal integration to detect the worm and the need to increase temporal resolution to catch it. The trade-off in this case is strictly determined by the kinetics of the dark-adapted rod responses, as shown in experiments where the rod kinetics was changed by warming or cooling and worm speed was varied. Warm toads were unable to benefit from the longer exposures afforded by slower worms; instead, they snapped more accurately than cool toads at the worms they did detect (Haldin et al., 2009).

Motion prediction and extrapolation probably occur universally in active animals. One widely used strategy for interception is maintaining a constant bearing angle (CBA) to the target, which is appropriate when its movements are not too erratic. CBA is applied, for example, by dragonflies capturing flying insects (Olberg et al., 2000) and humans catching flying balls (Diaz et al., 2009). It is typically complemented by other interception strategies, e.g. for balls moving towards the player (Michaels and Oudejans, 1992; Fink et al., 2009). Robber flies approach flying prey from below, maintaining CBA at longer distances, but switch to proactive flight locked on to the target at short range (Wardill et al., 2017). An especially striking, purely perceptual expression of neural motion extrapolation is the flash–lag effect (Nijhawan, 2002; Khoei et al., 2017): a brief light flash emitted by a moving object is perceived as lagging behind the object. Such illusions reveal how the neural mechanisms for motion prediction assume continuities in the physical world and thereby relax requirements on the speed of primary visual responses and processing capacity in tracking, interception or avoidance of moving objects. Whether targeting prey, predators or mating partners, these include some of the biologically most important tasks of temporal vision.

## Conclusions

Photoreceptor responses, which define the temporal information available for vision, are now reasonably well understood, as are their basic relationships with several simple temporal measures (TMTF, CFF, integration time, reaction time, temporal order). There is also increasing insight about temporal aspects of retinal processing, especially for motion vision. The present Review may be regarded as a ‘primal sketch’ of a research field with many unresolved questions. (1) Which aspects of temporal processing in different types of retinal cells really depend on the kinetics of the photoreceptor responses? One way to study this would be to do comparable experiments and modelling in conditions with different photoreceptor response kinetics, varied by, for example, light/dark adaptation, temperature or genetic modification. (2) What are the limits to motion discrimination by mammals in very dim light? What is the role of rod noise in motion detection near the absolute visual sensitivity limit? (3) How is temporal performance (in any interesting context) affected by noise? One way of studying this experimentally would be by adding calibrated temporal or spatio-temporal noise to the light stimuli – still an underexploited rationale in both electrophysiology and psychophysics. It is widely useful and can provide clear predictions, e.g. related to models based on temporal correlations. (4) What are the temporal implications, in different tasks, of parallel processing by tens of different GC types in the retinas of mice, humans and other vertebrates? How is temporal information from the ON and OFF pathways integrated? (5) How are things done in non-mammals? The most basic principles may be generalized across vertebrates and even across all seeing animals, but more complex neural operations diverge, and the relationship of homologies and analogies in animals viewing a common world is endlessly fascinating for evolutionary neuroscience and ecology.
